# Recent progress in H_2_S activated diagnosis and treatment agents

**DOI:** 10.1039/c9ra06698e

**Published:** 2019-10-18

**Authors:** Xiaodong Wang, Lu An, Qiwei Tian, Kuili Cui

**Affiliations:** The Key Laboratory of Resource Chemistry of the Ministry of Education, The Shanghai Key Laboratory of Rare Earth Functional Materials, The Shanghai Municipal Education Committee Key Laboratory of Molecular Imaging Probes and Sensors, Shanghai Normal University Shanghai 200234 China qiweitian@shnu.edu.cn anlu1987@shnu.edu.cn; Department of Tuberculosis, The First Affiliated Hospital of Xinxiang Medical University China

## Abstract

Hydrogen sulfide (H_2_S) is a key biosignal molecule in the human body. Endogenous H_2_S, as a gas delivery and protective agent in the body, is involved in a variety of physiological processes, including mediating vascular tone and neuromodulation. The production of abnormal H_2_S levels in the body is related to the occurrence of various diseases, so real-time monitoring of H_2_S *in vivo* is very important. However, traditional detection methods face enormous challenges in the *in vivo* detection of H_2_S owing to its high volatility and rapid catabolism. Optical probes developed in recent years with the advantages of high sensitivity, short response time, non-invasive nature and capacity for real-time monitoring can overcome the limitations of traditional detection methods and offer the possibility of real-time monitoring of H_2_S in cells and *in vivo*. In addition, the production of high concentrations of H_2_S is closely related to the formation of colon cancer, and H_2_S-activated treatment agents have been developed for use in this particular tumor microenvironment, which reduce the toxic side effects of traditional therapy on normal tissues and improves the treatment effect. This review summarizes the recent advances in H_2_S detection probes *in vitro* and *in vivo*, as well as H_2_S-activated tumor treatment agents.

## Abstract 2

This review summarizes the recent advances in H_2_S detection probes and H_2_S-activated tumor treatment agents.

## Graphical abstract

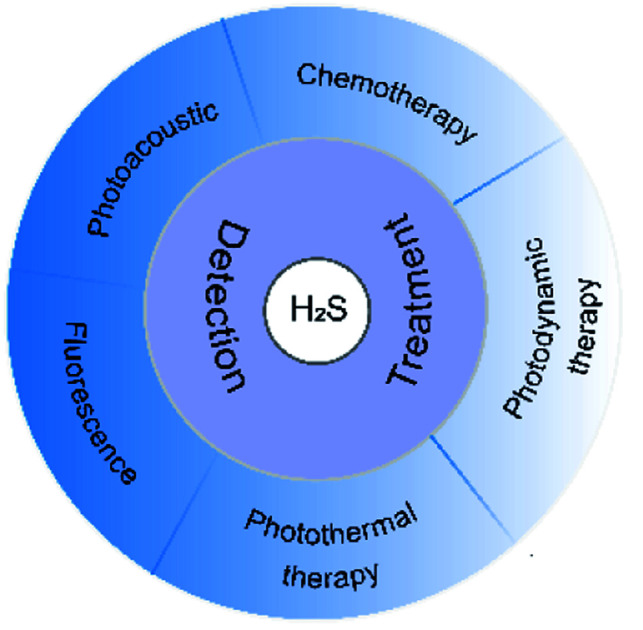

## 1. Introduction

Hydrogen sulfide (H_2_S) is an irritating gas with a smell of rotten eggs that has long been considered toxic.^1–4^ Recent studies have shown that H_2_S is an endogenously unstable gas, which has been identified as a gas carrier, as well as nitric oxide (NO) and carbon monoxide (CO).^5–7^ Endogenous H_2_S can be enzymatically produced by cystathionine γ-lyase (CSE), cystathionine β-synthase (CBS) and 3-mercaptopyruvate sulfurtransferase (3MST) in mammalian cells.^3,8–11^ These enzymes digest cysteine or cysteine derivatives and produce H_2_S in different organs. It has been shown that H_2_S is involved in many physiological processes,^12–14^ such as regulating blood pressure, exerting antioxidant and anti-inflammatory effects, and regulating the central nervous system,^15,16^ respiratory and gastrointestinal systems.^17^ The physiological concentration of H_2_S is 0.01–3 μM at the cellular level and 30–100 μM in the serum.^18^ Abnormal levels of H_2_S in the body can induce several malignant diseases, including Alzheimer's disease,^19^ diabetes, heart disease, hypertension and other cardiovascular diseases.^20^ Therefore, real-time detection of H_2_S levels is important for further study of its physiological and pathological roles in biological systems.

Traditional analytical methods for H_2_S mainly include colorimetry,^21^ electrochemical analysis,^22^ gas chromatography,^23^ and sulfide precipitation.^24^ These methods need high-standard preparation of samples and collection of H_2_S from cells or tissues.^25–27^ However, a fast H_2_S catabolism rate leads to fluctuations in its concentration, further resulting in inaccurate measurement.^28,29^ Therefore, the traditional methods have difficulty meeting fast, accurate, and real-time monitoring criteria for H_2_S levels in living systems. Optical detection methods are attracting increasing research interest owing to their high sensitivity, short response time, non-invasive nature, capacity for real-time monitoring and easy sample preparation.^30–33^ Based on the good nucleophilic and reducing chemistry of H_2_S, researchers have been developing optical probes with high sensitivity, selectivity and biocompatibility for the detection of H_2_S in biological systems. These probes are based primarily on specific H_2_S-induced reactions, including azide reduction,^34–36^ nitro reduction,^37,38^ removal of quenchers (such as copper(ii)),^39–41^ and nucleophilic reactions,^42–44^ to allow fluorescence to be turned on for H_2_S detection at different biological levels.

In addition, there have been some reports that CBS is selectively up-regulated and the concentration of H_2_S is significantly increased in cancer tissues such as colon, breast and ovarian cancers.^45–48^ H_2_S plays an important role in tumor proliferation and metastasis, and has become a new target for cancer treatment.^49^ Traditional cancer treatment methods mainly include surgical resection, chemotherapy, radiotherapy and other means.^50–52^ These treatment methods not only have a low cure rate, but also have relatively large side effects.^53^ Scientists are working to develop H_2_S-activated reagents for the treatment of cancer, on account of high concentrations of H_2_S in the tumor microenvironment. These mainly include: (i) H_2_S-activated nanodrug carriers for delivering chemotherapeutic drugs to tumor sites, improving the therapeutic efficiency of cancer while reducing the toxic side effects on normal tissues;^54^ (ii) H_2_S trapped in normal tissues after intravenous injection, causing damage to normal tissues on light irradiation. The H_2_S-activated phototherapy agent only produces therapeutic effects at the tumor site, thereby reducing damage to normal tissues.

In this review, we summarize the recent developments of H_2_S-activated probes in the biomedical field, including fluorescent probes and photoacoustic probes for *in vitro* and *in vivo* applications. In addition, the application and advantages of H_2_S-activated reagents in cancer diagnosis and treatment are also discussed. We also reference the side effects of traditional therapy reagents in the treatment of tumors, and describe the requirements and challenges of H_2_S-activated reagents. Finally, the possible future application prospects of H_2_S-activated diagnostic and therapeutic reagents for cancer therapy are also discussed.

## 2. H_2_S-activated probes

Abnormal H_2_S levels in organisms are associated with the development of many diseases.^15^ High-sensitivity probes for H_2_S concentrations in animals are very important; they can help us to understand the effects of H_2_S on various physiological and pathological processes, and to diagnose related diseases in a timely manner. Probes for H_2_S detection *in vitro*^55,56^ and *in vivo*^57–59^ are listed in Table 1 and described in detail below.

**Table 1:** Summary of recently published reports on H_2_S detection probes

H_2_S probe	Reaction mechanism	Wavelength (nm)	Detection limit	Experimental subject	Detection method	Ref.
Cyclen-AF + Cu^2+^ (HSip-1)	Cu^2+^ quenches fluorescence	516	10 μM	HeLa cells	Fluorescence microscopy	69
SHS-M2	Azides to amines	464/545	0.4 μM	DJ-1 deficient astrocytes and brain slices	Two-photon microscopy	71
NanoBODIPY	Nucleophilic reactions	511/589	7 nM	Raw 264.7 macrophage cells	Confocal microscopy	76
Coumarin–merocyanine dyad (CPC)	Nucleophilic reactions	474/587	40 nM	HeLa cells	Confocal microscopy	77
Azide-functionalized *O*-methylrhodol (MeRho-Az)	Azides to amines	516	86 ± 7 nM	C6 cells and zebrafish	Light sheet fluorescence microscopy	80
Ruthenium(ii) complex-based luminescence probe (Ru-MDB)	Nucleophilic reactions	456/612	45 nM	Zebrafish and mice	Fluorescence microscopy and confocal microscopy	83
NIR-II@Si	Nucleophilic reactions	*700/900*	37 nM	HCT116 tumor mice	Fluorescence imaging	85
Si@BODPA	Nucleophilic reactions	780	53 nM	HCT116 tumor mice	Photoacoustic imaging	88
AzHD-LP	Azides to amines	600/700	91 nM	HCT116 tumor mice	Photoacoustic imaging	89

### 2.1 H_2_S probes *in vitro*

H_2_S intelligent optical probes with high sensitivity, high selectivity, high signal-to-noise ratio and stability are being developed.^60^ Fluorescence imaging by fluorescent probe staining is one of the most attractive molecular imaging techniques for H_2_S detection in living cells, tissues and living animals.^61^ H_2_S-activated fluorescent probes are mainly based on the difference of emission wavelength before and after response.^62^ Although a lot of effort has been expended, fluorescence imaging is limited by problems such as the low concentration of endogenous H_2_S and the presence of a large number of interfering molecules, including reduced glutathione, cysteine (Cys) and thiol-containing proteins, in complex living systems. Therefore, it is still a significant challenge to develop highly sensitive and selective fluorescent probes.

Based on the nucleophilic and reductive properties of H_2_S, scientists have developed fluorescent probes for H_2_S detection founded on the reduction of azides to amines, nucleophilic reactions and copper sulfide precipitation.^63–66^ Liu *et al.*^67^ designed a H_2_S fluorescent probe containing bis-electrophile to take advantage the nucleophilicity of H_2_S. The fluorescence intensity of the disulfide-containing probe increased dramatically (55–70-fold) when 50 μM H_2_S was presented in solution. In addition, the maximum intensity was reached in 1 h, suggesting that the reaction was fast. The fluorescent probe is selective for H_2_S and does not react with other bio-thiols, such as cysteine and glutathione, at the same concentration (100 μM). A fluorophore of dansyl azide (DNS-Az) with high quantum yield was prepared by Peng *et al.*^68^ The azide is reduced to an amine by reduction with H_2_S to emit fluorescence for rapid detection of H_2_S *in vitro*. The probe was very sensitive, with a detection limit of 1 μM in buffer/Tween and 5 μM in bovine serum. The reaction was complete in a few seconds, while the fluorescence was enhanced immediately. No obvious response to the probe was observed for most of the tested anions at a concentration of 1 mM, which is a 40-fold higher concentration than that of sulfide. Sasakura *et al.*^69^ designed and synthesized a novel H_2_S-detecting fluorescent probe Cyclen-AF + Cu^2+^ (HSip-1) based on the azamacrocyclic ring to form a stable metal complex with Cu^2+^. The paramagnetic Cu^2+^ center could quench the fluorophore's fluorescence. When H_2_S binds to Cu^2+^, Cu^2+^ is released from the azamacrocyclic ring, resulting in enhanced fluorescence. The probe showed a large (50-fold) and immediate increase in the fluorescence intensity upon addition of 10 μM H_2_S, whereas almost no fluorescence increment was observed upon the addition of 10 mM GSH. Thus, HSip-1 is more selective for H_2_S than previously reported fluorescent probes using 2,4-dinitrosulfonyl or azide groups.

**Fig. 1:**
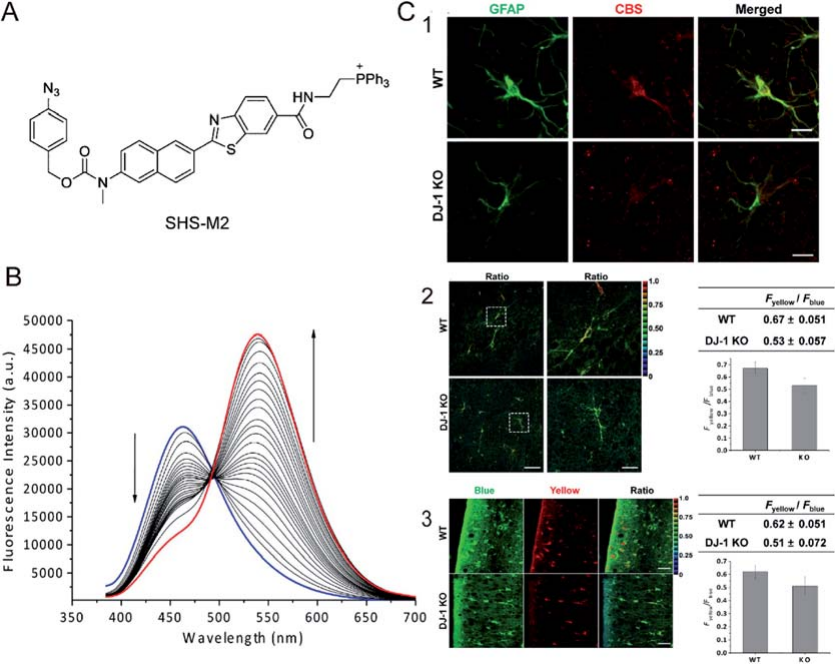
(A) The structure of SHS-M2. (B) Fluorescence response of 1 μM SHS-M2 to 100 μM Na_2_S in HEPES buffer from 0 to 60 min. *λ*_ex_ = 373 nm. (C) The relationship between CBS expression and H_2_S production in astrocytes of DJ-1 knockout (KO) brain. Brain slices were prepared from wild-type (WT) and DJ-1 KO mice. (1) Hippocampal slices were prepared and stained for GFAP (an astrocyte marker) and CBS-expressed H_2_S; (2) H_2_S analysis of freshly prepared slices; (3) cortical slices were cultured for 7 days after slicing to stabilize the tissues from slicing stress, and then the H_2_S production was measured. Reproduced from ref. 71. Copyright 2013 American Chemical Society.

For most single-window-response fluorescent probes the experimental results change with the experimental conditions.^70^ Ratiometric fluorescent probes are able to overcome the interference due to experimental conditions. Bae *et al.*^71^ reported a H_2_S-activated mitochondrially localized two-photon ratiometric fluorescent probe, SHS-M2 (Fig. 1A), which has 6-(benzo[*d*]thiazol-2-yl)-2-(methylamino) naphthalene as the fluorophore, 4-azidobenzyl carbamate as the H_2_S response site, and triphenylphosphonium salt as the mitochondria-targeting moiety. The thiolate-triggered reaction with the azide group would cleave the carbamate linkage and liberate the amino group, accompanied by a decrease in emission intensity at 420 nm and a gradual increase at 500 nm. The color also changes from blue to yellow. Thereby, the emission and the cross-section of the ratiometric two-photon probe can be increased (Fig. 1B). The probe is more sensitive and the detection limit of H_2_S is 0.4 μM *in vitro*. The fluorescence intensity of the SHS-M2 after triggering by H_2_S (0.1 mM) is 5–8-fold higher than that with 10 mM glutathione (GSH) and 1 mM cysteine (Cys), which confirms the high selectivity for H_2_S over GSH and Cys. Two-photon microscopy ratiometric imaging of SHS-M2 as a probe can be used to study the relationship between CBS expression and H_2_S levels in cells and brain sections (Fig. 1C).

**Fig. 2:**
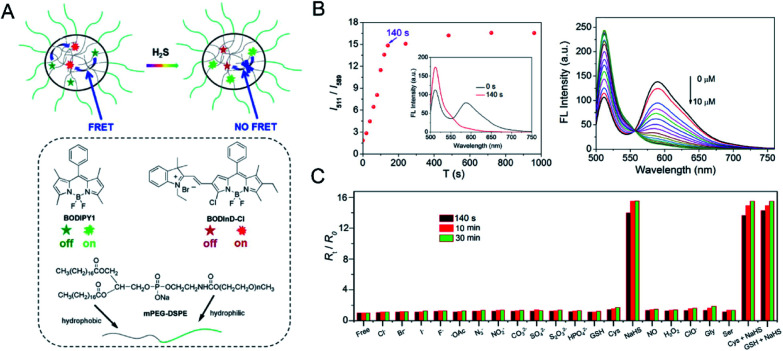
(A) The structure of NanoBODIPY and the FRET process from the complementary energy donor (BODIPY1) to the responsive energy acceptor BODInD-Cl. (B) Change in ratiometric fluorescence intensity of NanoBODIPY in the presence of NaHS (100 μM) at different times and fluorescence spectra in various concentrations of NaHS (0, 0.5, 1.0, 1.5, 2.0, 2.5, 3.0, 3.5, 4.0, 4.5, 5.0, 5.5, 6.0, 6.5, 7.0, 7.5, 8.0, 8.5, 9.0, 9.5, 10.0 μM, respectively). (C) Ratiometric fluorescence changes of NanoBODIPY in the presence of 100 μM NaHS and other biologically relevant competing analytes. *λ*_ex_ = 490 nm. Reproduced from ref. 76. Copyright 2015 American Chemical Society.

The main problem of current H_2_S probes is low detection sensitivity. Förster resonance energy transfer (FRET)-based fluorescent probes can eliminate the effect of excitation backscattering on fluorescence detection because of the large offset between donor excitation and acceptor emission.^72,73^ In addition, two well-separated emission bands with comparable intensities can be used to ensure the accuracy of their strength and ratio. Some fast and accurate ratiometric fluorescent probes for detecting H_2_S have been developed based on FRET.^74,75^ Zhao *et al.*^76^ developed a self-assembled micelle aggregate NanoBODIPY fluorescent probe with H_2_S-triggered FRET switch, which consists of a dynamic energy receptor semi-cyanine-BODIPY hybrid dye (BODInD-Cl) and a complementary energy donor (BODIPY1). In the absence of H_2_S, a specific FRET from BODIPY1 to BODInD-Cl occurs due to the spectral overlap between the emission spectrum of the donor and the absorption spectrum of the acceptor. In contrast, in the presence of H_2_S, the Cl on the aromatic ring in NanoBODIPY is replaced by the H_2_S *via* nucleophilic substitution and the absorption of the probe is shifted from 540 to 738 nm, resulting in loss of FRET owing to the lack of overlap between the emission spectrum of the donor and the absorption spectrum of the acceptor (Fig. 2A). This results in a fluorescence signal that simultaneously “turns on” the energy donor BODIPY1 and a fluorescence signal that “closes” the energy acceptor BODInD-Cl. NanoBODIPY can sensitively and quickly detect H_2_S with a detection limit of 7 nM by ratiometric fluorescence. The emission intensity gradually increased at 511 nm after adding different concentrations of sodium hydrosulfide (NaHS), accompanied by a loss of emission at 589 nm, and the response was complete within 140 s (Fig. 2B). Through competitive experimental studies, NanoBODIPY showed good selectivity for NaHS with minimal interference from other biologically relevant analytes in PBS buffer (Fig. 2C).

By a similar approach, Feng *et al.*^77^ reported a FRET-based ratiometric fluorescent probe composed of a coumarin–merocyanine dyad. Before the reaction with H_2_S, the emission wavelength of coumarin apparently overlaps with the absorption of merocyanine, and a resonance energy transfer process occurs, so that the probe displays the fluorescence of the cyanine. In the presence of H_2_S, the merocyanine moiety undergoes a nucleophilic addition reaction with H_2_S, and the conjugated system is destroyed; as a result, resonance energy transfer cannot be achieved, and so the fluorescence of coumarin is exhibited. The probe has a detection limit of as low as 40 nM. It can be used for mitochondrial endogenous and exogenous H_2_S detection; it shows a greater emission shift than other H_2_S probes, and so it exhibits higher selectivity and sensitivity.

### 2.2 H_2_S probes *in vivo*

Despite rapid progress in the development of H_2_S probes in the past few years, there are still many problems in the transition from solutions, cells and tissues to whole organisms. Tissue penetration, poor spatial resolution in deep biological tissues, fluorophore stability at high excitation wavelengths and other issues have largely limited their application for *in vivo* H_2_S detection.

#### 2.2.1 Fluorescent probes

**Fig. 3:**
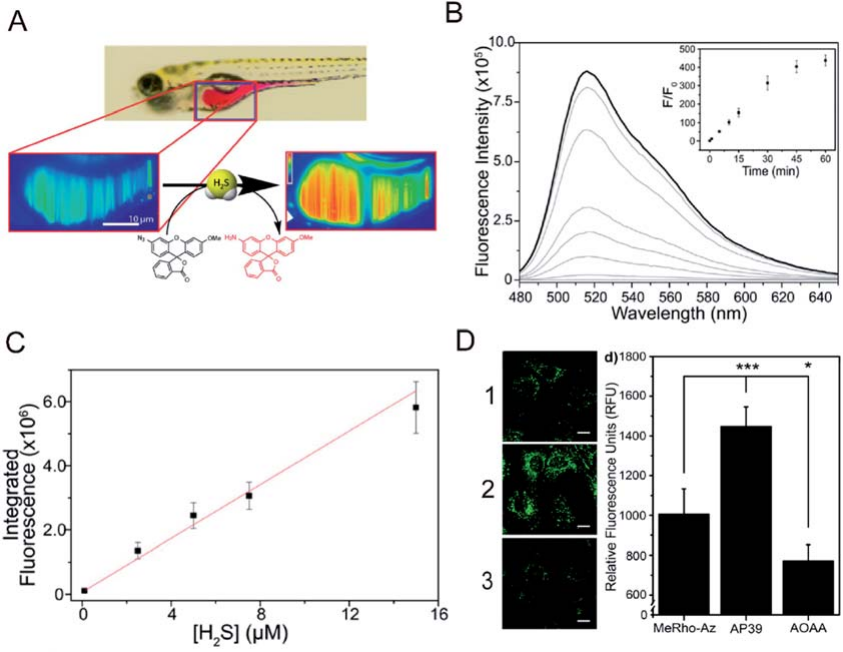
(A) Schematic of 3D imaging in live zebrafish using light sheet fluorescence microscopy. (B) Uncorrected fluorescence spectra response of 5 μM MeRho-Az to 250 μM NaHS treatment over 60 min. *λ*_ex_ = 476 nm, *λ*_em_ = 480–650 nm. (C) Fluorescence intensity of MeRho-Az in the presence of various concentrations of NaHS for 90 min. (D) Fluorescence imaging of H_2_S in C6 cells treated with (1) MeRho-Az probe, (2) AP39 (H_2_S donor) and (3) AOAA (H_2_S inhibitor) respectively. Reproduced from ref. 80. Copyright 2015 American Chemical Society.

The light sheet fluorescence microscope (LSFM) is an imaging tool that confines excitation light to a sheet that coincides with the focal plane of a wide field of view imaging system.^78^ The LSFM can image larger samples than confocal microscopes while enabling rapid imaging. The LSFM combined with a H_2_S-responsive fluorescent probe enables detection of H_2_S levels *in vivo*.^79^ Hammers *et al.*^80^ developed an azide-functionalized *O*-methylrhodol fluorophore (MeRho-Az) for the detection of H_2_S in live zebrafish (Fig. 3A). The xanthene core modified *O*-methylrhodol (MeRho) is locked in the non-fluorescent spirolactone tautomeric form. The H_2_S reduction of azide regenerates the amine while releasing the fluorescent open tautomer to produce an intense fluorescence, and exhibits a rapid >1000-fold fluorescence response. MeRho-Az can sensitively detect low concentrations of H_2_S, with a detection limit of 86 ± 7 nM. Owing to the pH insensitivity and photostability of MeRho-Az, it can be used for the detection of H_2_S in living organisms. The results showed that the fluorescence signal was rapidly increased after the addition of NaHS to MeRho-Az (Fig. 3B and C). Then MeRho-Az was used to detect endogenous H_2_S in C6 rat glial cells by fluorescence imaging. The fluorescence signal of the C6 rat glial cells in the group treated with AP39 (H_2_S donor) plus MeRho-Az was higher than that for the group treated with MeRho-Az alone. In contrast, the fluorescence signal of the group treated with AOAA (aminooxyacetic acid; H_2_S inhibitor) plus MeRho-Az was lower than that of the group treated with MeRho-Az alone. These results demonstrated that MeRho-Az can sensitively detect low concentrations of H_2_S (Fig. 3D).

**Fig. 4:**
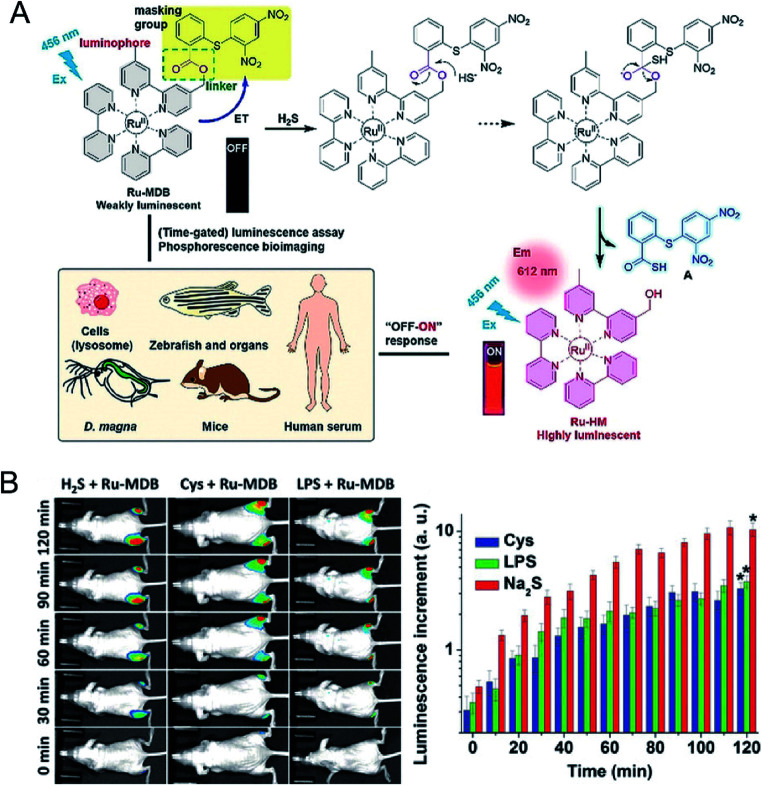
(A) Strategy for the design and phosphorescence response mechanism with H_2_S of Ru-MDB, and the application of Ru-MDB in quantitative monitoring and visualizing of H_2_S *in vitro* and *in vivo*. (B) Luminescence imaging of H_2_S in live mice using Ru-MDB as a probe, and time-dependent increments of mean luminescence intensities. One group had Ru-MDB subcutaneously injected into the left and right hindlegs, followed by the injection of H_2_S into the left hindleg and the imaging of the mice at different times. In the other two groups, cysteine (Cys) and lipopolysaccharide (LPS), respectively, were injected into the right hindleg, and then Ru-MDB was injected into the left and right hindlegs. Reproduced from ref. 83. Copyright 2018 WILEY-VCH.

Phosphorescent transition metal complexes have attracted much attention owing to their strong visible light absorption and emission, large Stokes shift, and stable photochemical properties.^81,82^ A ruthenium(ii) complex-based responsive luminescence probe (Ru-MDB) for H_2_S detection was studied by Du *et al.*^83^ MBD is a masking moiety for the Ru-MDB complex H_2_S response. The metal-to-ligand charge transfer (MLCT) excited state of the Ru^II^ complex is destroyed by an intramolecular light-induced electron transfer photo-induced electron transfer (PET) process when the electron acceptor group MDB is linked (Fig. 4A). To utilize the nucleophilic properties of H_2_S, the new MDB masking group was linked to one of the bipyridine ligands of the Ru^II^ complex through an ester bond that could be cleaved by H_2_S, resulting in an approximately 86-fold increase in luminescence intensity. The detection limit was measured to be 45 nM, which suggested high sensitivity of Ru-MDB for monitoring H_2_S in mice. The main characteristics of this probe enabled the monitoring of lysosomal H_2_S generation in live cells, and the visualization of exogenous/endogenous H_2_S in live *Daphnia magna*, zebrafish and mice (Fig. 4B).

**Fig. 5:**
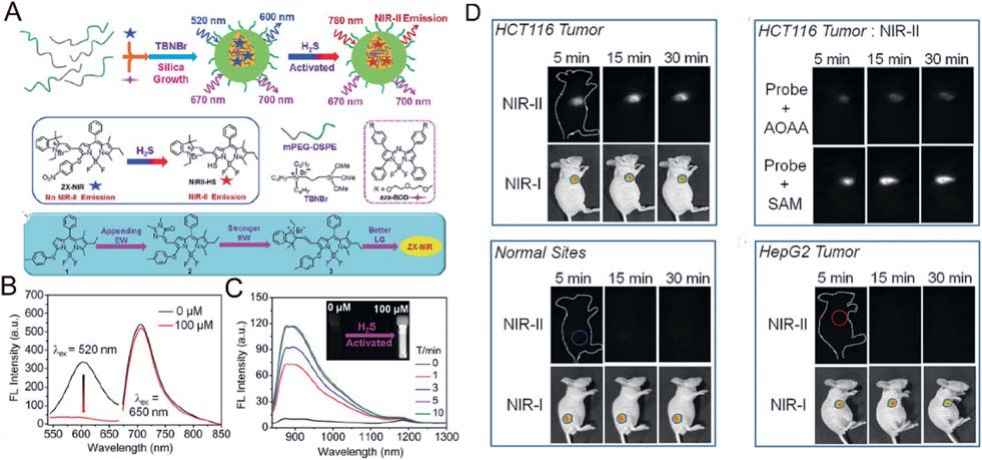
(A) Schematic of the construction of multi-wavelength nanoprobes with activatable emission in the second near-infrared (NIR-II) window. (B) Fluorescence changes of NIR-II@Si (10 μM ZX-NIR) upon addition of 100 μM NaHS in PBS (pH 7.4). (C) Time-dependent NIR-II emission spectra. Inset: photograph of the H_2_S-activated NIR-II emission. (D) *In vivo* fluorescence imaging of mice bearing two different tumor types using H_2_S-activated NIR-II@Si nanoprobe. Reproduced from ref. 85. Copyright 2018 WILEY-VCH.

Fluorescence imaging in the second near-infrared window (NIR-II, 1000–1700 nm) showed reduced autofluorescence, enhanced tissue penetration, and higher spatial resolution *in vivo*.^84^ Xu *et al.*^85^ designed a H_2_S-activated NIR-II@Si fluorescent probe (Fig. 5A) that visualizes colorectal cancer. The probe encapsulates the H_2_S-responsive fluorescent probe in the hydrophobic interior of the core–shell silica nanocomposite. The fluorescent nanoprobes comprise two organic chromophores: boron-dipyrromethene (ZX-NIR) dye, which has a maximum emission shift from 600 nm to 900 nm in the presence of H_2_S to produce NIR-II emission, and aza-BODIPY (aza-BOD), the emission of which remains unchanged at 700 nm, as an internal reference (Fig. 5B and C).The detection limit for H_2_S was measured to be 37 nM, indicating the high sensitivity of NIR-II@Si for ratiometric detection of H_2_S. This activatable H_2_S-specific targeting probe can be used for deep tissue imaging of H_2_S-rich colon cancer cells. Utilizing the advantages of NIR-II imaging, tumor sites can be selectively detected, and visual monitoring of tumor models of colon cancer can be achieved (Fig. 5D).

#### 2.2.2 Photoacoustic probes

Fluorescence imaging is limited by problems such as poor tissue penetration and autofluorescence, and few probes can be used for imaging in deep tissues and whole animals. In order to solve these problems, it is highly desirable to develop a probe with a new mode of imaging. Photoacoustic imaging combines the advantages of the high resolution of optical imaging and high penetration depth of ultrasound imaging.^86,87^ It is a medical imaging diagnostic technology with broad clinical application prospects.

**Fig. 6:**
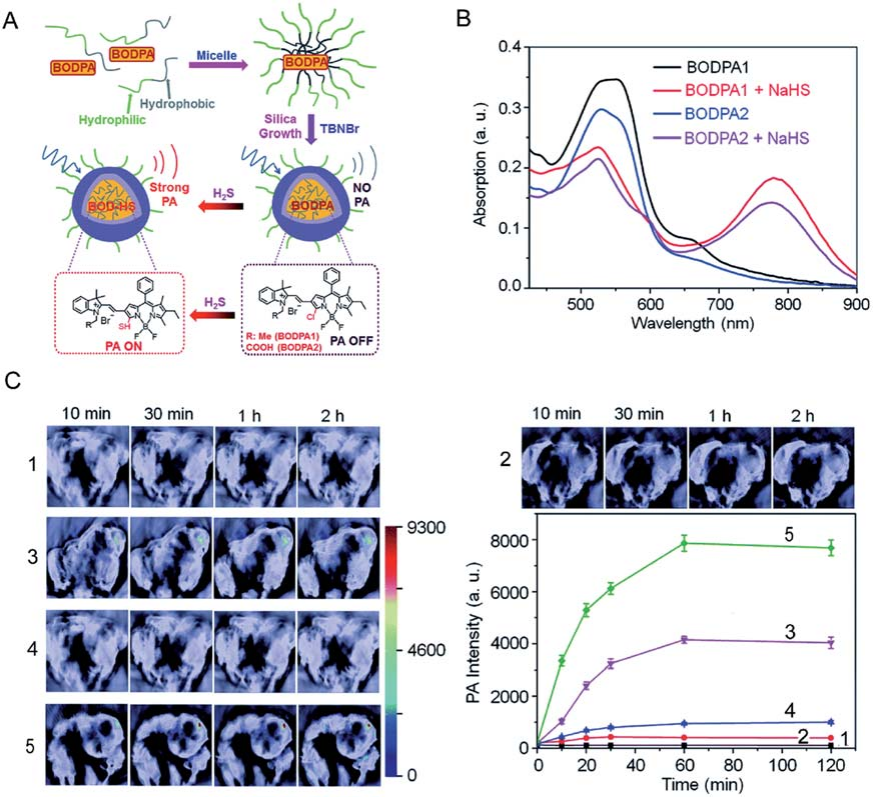
(A) Schematic illustration of the construction of activatable photoacoustic probes for H_2_S. (B) The absorption changes of Si@BODPAs (10 μM BODPA1 or BODPA2) in the absence and presence of NaHS (100 μM). (C) Photoacoustic imaging of tumor-bearing mice using Si@BODPA1 at different times: (1) saline-treated mice in the tumor regions; (2) probe-treated mice at normal sites; (3) probe-treated mice in the tumor regions; (4,5) mice pre-treated with (4) 100 nmol AOAA, or (5) 300 nmol *S*-adenosyl-l-methionine for 12 h, were subcutaneously injected with Si@BODPA in the tumor regions. Graph, photoacoustic intensities as a function of time post-injection of Si@BODPA. Reproduced from ref. 88. Copyright 2017 Royal Society of Chemistry.

In the last few years, people in related fields have been working on developing photoacoustic probes for detecting H_2_S *in vivo*. Shi *et al.*^88^ developed a H_2_S-activated Si@BODPA photoacoustic probe that encapsulates a semi-cyanine-BODIPY hybrid dye (BODPA) in the interior of a silica nanocomposite (Fig. 6A); thereby the probe has good water solubility and excellent biocompatibility. Conversion of BODPA to BOD-HS within the nanoparticles (NPs) by aromatic nucleophilic substitution in the presence of H_2_S results in high NIR absorption around 780 nm (Fig. 6B). Therefore, the Si@BODPA probe produces a strong photoacoustic signal output in the NIR region. The detection limit was measured to be 53 nM. The probe shows an extremely fast response and can detect transient changes in H_2_S. Si@BODPA allows direct photoacoustic tracking of endogenous H_2_S production in an HCT116 (human colon cancer cell) tumor-bearing mouse model. As shown in Fig. 6C, there was no photoacoustic signal from the normal sites of mice and the tumor site of the mice pre-treated with the CBS inhibitor aminooxyacetic acid (AOAA, 100 nmol) after injection of Si@BODPA, while a photoacoustic signal was observed in the tumors of the mice without and with pretreatment with a CBS activator (*S*-adenosyl-l-methionine), indicating that Si@BODPA can be used for detection of H_2_S *in vivo*.

**Fig. 7:**
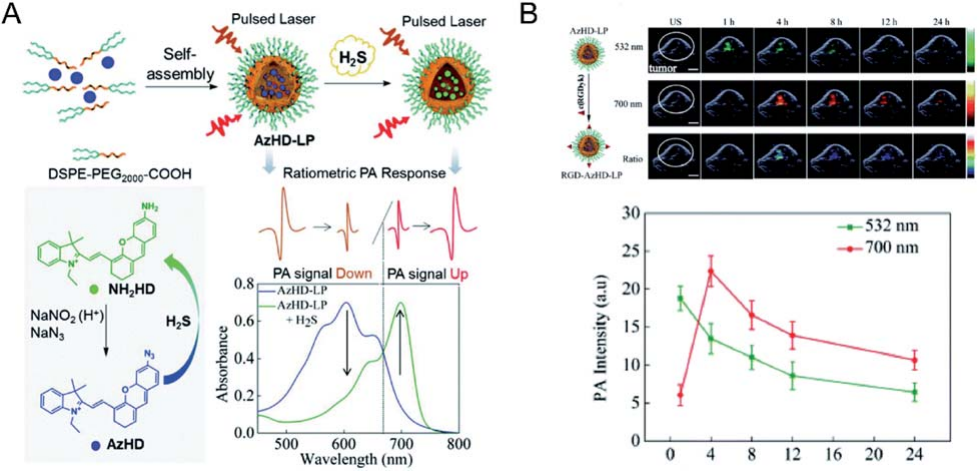
(A) Illustration of the construction of activatable nanoprobe AzHD-LP and the proposed mechanism for ratiometric photoacoustic (PA) detection of H_2_S. (B) PA/ultrasound (US) overlaid images of subcutaneous HCT116 tumor in naked mice pretreated with RGD-AzHD-LP, and plot of ratiometric intensity (PA_700_/PA_532_) against time. Reproduced from ref. 89. Copyright 2018 Royal Society of Chemistry.

At present, photoacoustic probes for H_2_S detection mostly provide single-response photoacoustic signals, and the results will be affected by factors such as instrument, probe concentration and external environment. On the contrary, a ratiometric photoacoustic probe can eliminate the effects of the above factors by using the ratio of two separate wavelength photoacoustic response signals, thereby obtaining reliable experimental results. Ma *et al.*^89^ developed a novel ratiometric photoacoustic nanoprobe for *in vivo* detection of H_2_S. The nanoprobe AzHD-LP consists of a liposome (LP) with a H_2_S-responsive near-infrared dye (AzHD) encapsulated inside it (Fig. 7A). After the reduction of azide to amine in the AzHD-LP photoacoustic probe by H_2_S, the absorption peak appears red-shifted. The absorption of AzHD-LP at 600 nm is reduced, while the absorption at 700 nm is increased, resulting in a ratiometric PA signal in the presence of H_2_S. The detection limit of AzHD-LP for NaHS in solution was determined to be 91 nM. Furthermore, after AzHD-LP was conjugated to tumor-targeting peptide c(RGDyK), detection of intratumoral H_2_S production in HCT116 colon tumor mice was achieved under excitation of 532 nm and 700 nm pulsed lasers (Fig. 7B).

In this section, fluorescent probes and photoacoustic (PA) probes for H_2_S detection are introduced. Although fluorescent probes are widely used in the detection of H_2_S, their applications *in vivo* are limited by the autofluorescence and penetration depth. Photoacoustic imaging with high tissue penetration can be used to detect H_2_S levels in the living body and accurately locate a lesion. However, their sensitivity impedes their further application. As a result, it is necessary to develop better probes. NIR-II fluorescence and NIR-II photoacoustic imaging^90,91^ are emerging technologies that exhibit greater penetration depth and higher sensitivity. Therefore, the design of NIR-II fluorescence probes with weaker autofluorescence and NIR-II PA probes is the way forward.

## 3. H_2_S-activated therapeutic reagents

Compared with the traditional treatment of colon cancer, targeted response therapy can reduce side effects and cause more obvious therapeutic effect. Overexpression of cystathionine-β-synthase (CBS) in tumor cells leads to an increase in H_2_S levels (0.3 to 3.4 mM), especially in colon tumor cells.^45^ So, it will be more efficient to use H_2_S-activated therapy for colon cancer than other tumor microenvironment factors (pH, GSH, *etc*). Therefore, a series of H_2_S-activated therapeutic reagents have been designed on account of endogenous hydrogen sulfide, which is highly expressed in colon tumors, including H_2_S-activated chemotherapy, photodynamic therapy, and photothermal therapy (Table 2).

**Table 2:** Summary of recent reports on H_2_S therapeutic agents

Therapeutic agent type	Therapeutic strategy	Tumor species	Ref.
Mesoporous silica nanoparticle (MANP-N_3_-FA)	Chemotherapy	Colon cancer	94
N_3_-Nap-PHEMA-*b*-PMMA-N_3_	Chemotherapy	Cervical cancer	95
CuDOX NP	Chemotherapy	Cervical cancer	96
[Cu_2_(ZnTcpp)·H_2_O]_*n*_	Photodynamic therapy	Colon cancer	99
Electrochromic materials (EMs)	Photodynamic therapy	Colon cancer	100
Theranostic prodrug (Nano-TNP-SO)	Photodynamic therapy	Colon cancer	101
Self-assembled H_2_S response small molecule (SSS)	Photothermal therapy	Colon cancer	103
Cu_2_O	Photothermal therapy	Colon cancer	104

### 3.1 Chemotherapy

**Fig. 8:**
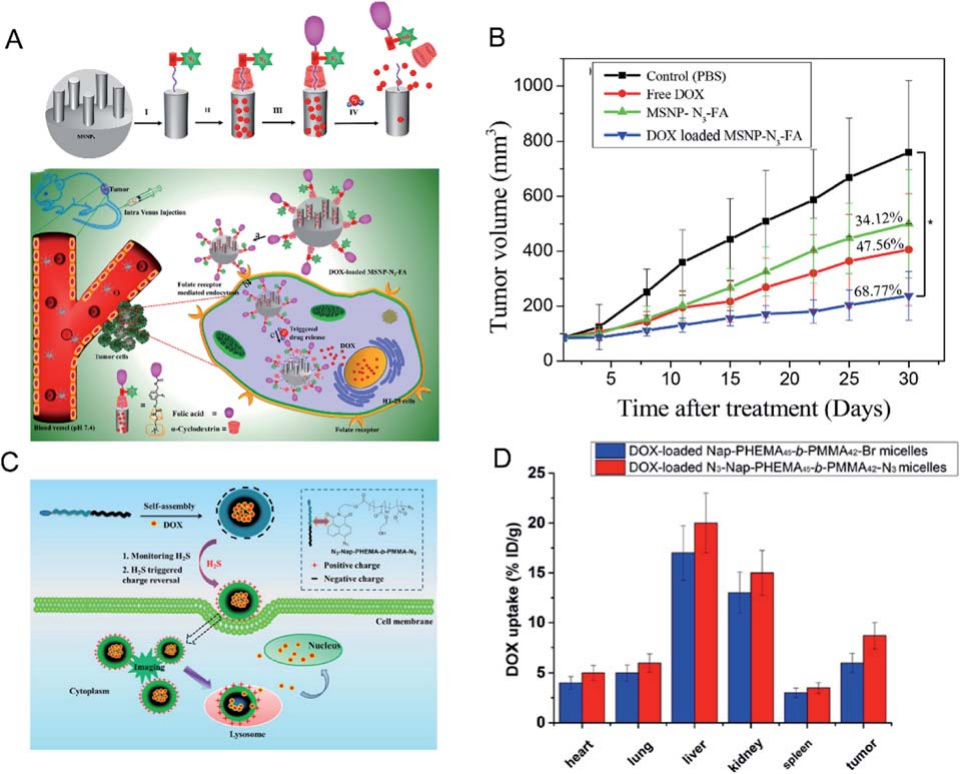
(A) Surface functionalization on MSNPs and mobilization of DOX-loaded MSNP-N_3_-FA into HT-29 cells, and H_2_S-triggered drug release inside the cell. (B) Antitumor efficacy of DOX-loaded MANP-N_3_-FA *in vivo*. Reproduced from ref. 94. Copyright 2019 American Chemical Society. (C) Schematic illustration of how H_2_S triggers charge reversal and cellular uptake of N_3_-NapPHEMA_45_-*b*-PMMA_42_-N_3_ micelles. (D) Biodistribution of DOX in 4T1 tumor-bearing mice at 4 h post-injection. Data are presented as percentage of injected dose per gram (%ID per g). Reproduced from ref. 95. Copyright 2016 American Chemical Society.

Chemotherapy is currently the main method used in the clinical treatment of cancer. Current chemical drugs for cancer treatment include doxorubicin (DOX),^92^ curcumin^93^ and so on. Unfortunately, we have not yet broken through the bottleneck in finding chemical drugs with excellent anti-tumor effects. Since most chemotherapeutic drugs have poor water solubility and low bioavailability, systemic administration is very difficult. The key problem is that normal cells will be damaged when the drugs are administered intravenously, resulting in toxic side effects. Therefore, scientists have long desired to develop a drug carrier from which the release of chemotherapeutic drugs can be stimulated at the tumor site only. In order to increase the targeting effect on tumor tissues and improve the therapeutic effect, a hydrogen sulfide-activated azide-functionalized biocompatible mesoporous silica nanoparticle (MSNP) was developed by Thirumalaivasan *et al.*^94^ as a specific drug delivery system (Fig. 8A). Further, folic acid (FA) was attached to the surface of the MSNP to actively target cancer cells. In the presence of H_2_S, the ester bond in the DOX-loaded MSNP-N_3_-FA is cleaved, resulting in the release of DOX from the MSNP, while no DOX is released from the MSNP before being activated by H_2_S. The *in vivo* results based on HT-29 tumor mice suggested that the therapeutic effect of MSNP-N_3_-FA with DOX is greater than that of DOX or MSNP-N_3_-FA alone (Fig. 8B).

Similarly, Zhang *et al.*^95^ used a series of *N*-(2-hydroxyethyl)-4-azide-1,8-naphthalimide-ended amphiphilic diblock copolymer poly(2-hydroxyethyl methacrylate)-*block*-polymethylmethacrylate (N_3_-Nap-PHEMA-*b*-PMMA-N_3_) polymer nano-micelles for loading DOX (Fig. 8C). Under the action of H_2_S, the charge on the surface of the micelles of these nanomaterials is reversed and the azide reduction reaction occurs. The surface charge of the micelles changes from negative to positive, which promotes the uptake of the materials by the cells and accelerates the release of DOX (Fig. 8D).

A pharmaceutical carrier should have excellent biocompatibility. Chen *et al.*^96^ designed a H_2_S-activated protein cage (CuDOX NP) loaded with chemotherapeutic drugs. They used horse spleen apoferritin (apo-HSF) as a container for copper-complexed doxorubicin to obtain a water-soluble nanocomposite. Breaking of the CuDOX coordination interaction by H_2_S under physiological pH conditions allows the DOX to be slowly released from the protein cage without disrupting the structure of the protein. *In vitro* cell experiments showed that CuDOX nanoparticles activated by H_2_S can reduce the premature release of drugs, reduce the toxicity of DOX to normal cells, and enhance the anti-cancer effect.

### 3.2 Photodynamic therapy

**Fig. 9:**
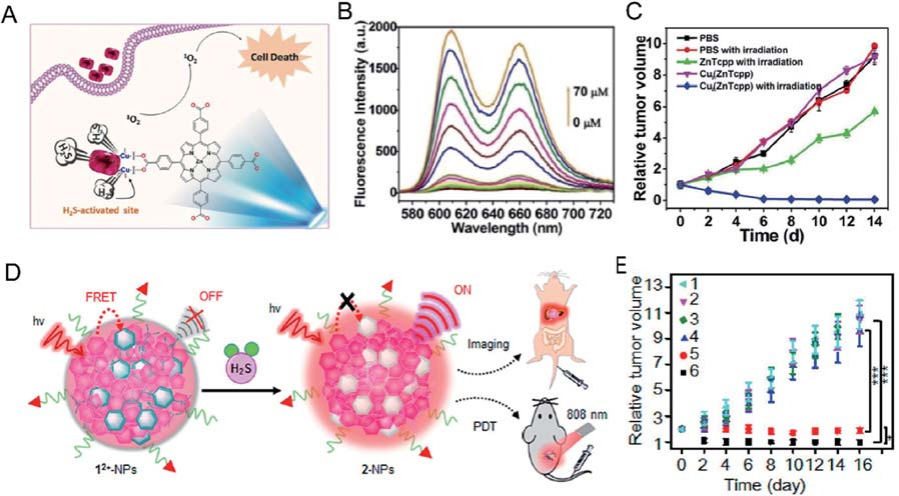
(A) The simple structural fragment of MOF NP-1 and the proposed strategy for ^1^O_2_ generation in cancer therapy. (B) Fluorescence spectra of NP-1 reaction with HS^−^ from 0 to 70 μM. (C) *In vivo* antitumor efficacy of NP-1 on HCT116 subcutaneous xenograft nude mice. Reproduced from ref. 99. Copyright 2017 WILEY-VCH. (D) Schematic illustration of H_2_S-activatable 1^2+^-PSs-FA enabling controllable ^1^O_2_ generation for PDT. (E) The tumor treatment effect of 1^2+^-PSs-FA. Reproduced from ref. 100. Copyright 2018 American Chemical Society.

Photodynamic therapy (PDT) is based primarily on the accumulation of non-toxic photosensitizers, oxygen and light to produce reactive oxygen species, particularly singlet oxygen (^1^O_2_), which selectively induces apoptosis and necrosis in cancer cells.^97,98^ PDT serves as a specific method for treatment of cancer because of its multiple merits, including non-invasiveness, obvious therapeutic effect, and lack of inhibition and adverse effects on the host system. However, most PDT agents are extremely hydrophobic, easily aggregate in aqueous solution, and have low accumulation in cancerous tissues, resulting in less generation of ^1^O_2_ at the required site. Moreover, they are easily trapped in normal tissues, and damage normal tissues during treatment. Therefore, it is worthwhile to develop intelligent photosensitizer agents (PSs) with good hydrophilicity that selectively accumulate at the tumor site. Effective tumor photodynamic therapy could be achieved by exploiting the high expression of endogenous H_2_S in colon cancer using a photosensitizer that recovers fluorescence under the activation of H_2_S. Ma *et al.*^99^ reported a nanoscale copper–zinc mixed-metal organic framework photosensitizer, [Cu_2_(ZnTcpp)·H_2_O]_*n*_ (NP-1), activated by H_2_S for photodynamic therapy of colon cancer (Fig. 9A). 5,10,15,20-Tetrakis(4-methoxycarbonylphenyl)porphyrin (ZnTcpp) is a bridged photosensitive ligand with a mixed metal organic skeleton in which Cu^2+^ ions serve as a metal node of the skeleton. The paramagnetic Cu^2+^ ions not only completely quench the ligand fluorescence of the metal–organic framework (MOF) NPs, but also significantly reduce release of reactive oxygen species (ROS). H_2_S interacts with [Cu_2_(ZnTcpp)·H_2_O]_*n*_, and Cu^2+^ ions are taken out from the MOF node to obtain a photosensitizer, and the fluorescence is recovered (Fig. 9B). This open-type fluorescent MOF photosensitizer probe achieves effective cancer treatment through controlled release of photoactive ligands, and the experimental results showed significant therapeutic effects (Fig. 9C).

In addition, Wu *et al.*^100^ reported a class of H_2_S-activated fluorescent probes and photodynamic smart reagents using electrochromic materials (EMs) with organic π-electron structure (dicationic 1,1,4,4-tetraphenylbutadiene, 1^2+^) as H_2_S-responsive chromophores. EM1^2+^ is doped into semiconductor polymer nanoparticles (SNPs) to form H_2_S-activatable fluorescent probes (1^2+^-SNPs) (Fig. 9D). Within 1^2+^-SNPs, EM1^2+^ can effectively quench the fluorescence of SNP by a fluorescence resonance energy transfer (FRET) process. Subsequent reduction of 1^2+^ to colorless 2 NPs by H_2_S eliminates the FRET process and restores fluorescence. Further, tumor-targeting ligand folic acid modified fluorescent probes (1^2+^-SNP830-FA) were used for tumor imaging in H_2_S-enriched mice. Tumor-targeting and H_2_S-activatable PSs (1^2+^-PSs-FA) using EM1^2+^ were further developed by replacing the SNP with organic PS. 1^2+^-PSs-FA accumulates well at the tumor site. After H_2_S-specific activation, 1^2+^-PSs-FA produces ROS under the action of 808 nm laser irradiation. The reagent exhibits negligible phototoxicity to normal tissues and significant tumor photodynamic therapy effects (Fig. 9E).

In addition, Wang *et al.*^101^ have designed and synthesized a theranostic prodrug (TNP-SO) for H_2_S-activatable near-infrared emission-guided on-demand administration of PDT. The theranostic probe consists of an H_2_S-activated NIR imaging probe and a sensitizing drug. These two units are connected by a short diglycolamine spacer. The newly obtained small molecule probe is encapsulated into the hydrophobic interior of a silica nanocomposite to produce a nanoprobe with good water solubility and photostability. The absorption of TNP-SO at 509 nm decreased as 677 nm NIR absorption increased after being triggered by H_2_S. The NIR fluorescence increased linearly with H_2_S concentration (0–20 μM), and the determined detection limit was 21 nM, indicating that Nano-TNP-SO has high sensitivity for H_2_S detection. Nanoprobes can also act as good photosensitizers for the efficient production of ^1^O_2_. The *in vivo* results using this probe reveal that cancer imaging accurately guides the location of light exposure to produce the cytotoxic ROS required for on-demand cancer treatment, maximizing treatment efficiency and minimizing side effects.

### 3.3 Photothermal therapy

**Fig. 10:**
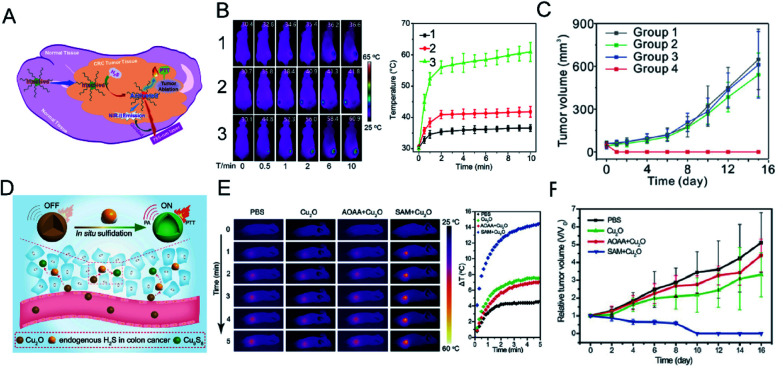
(A) Schematic diagram of photothermal activation for NIR-II fluorescence guidance treatment of colorectal cancer rich in H_2_S. (B) Infrared thermal images and heating effect in HCT116 tumor-bearing mice under continuous NIR laser irradiation and mean temperature as a function of irradiation time. (1) No administration of probes; (2) mice treated with probe at normal sites; (3) mice treated with probe in the tumor regions. (C) *In vivo* treatment results of photothermal therapy with Nano-PT. Reproduced from ref. 103. Copyright 2018 American Chemical Society. (D) Schematic diagram of Cu_2_O *in situ* reaction mechanism. (E) The temperature-increasing effect at the tumor site under laser irradiation (808 nm, 1 W cm^−2^). (F) *In vivo* photothermal therapy effect of Cu_2_O on HCT116 tumor. Reproduced from ref. 104. Copyright 2018 WILEY-VCH.

Photothermal therapy is a simple, safe, non-invasive treatment method that converts near-infrared laser energy into heat energy to achieve local high-temperature killing of tumor cells.^102^ Near-infrared photothermal reagents based on photothermal therapy have attracted much attention. Traditional photothermal reagents have limitations such as non-specificity and toxicity. In order to solve these problems, photothermal reagents with intelligent response are required. Shi *et al.*^103^ developed a H_2_S-activated second near-infrared self-assembling fluorescent nanoprobe for guiding photothermal therapy of colon cancer (Fig. 10A). A self-assembled H_2_S response small molecule (SSS) was designed that contains three triethylene glycol monomethyl ether chain functionalized benzene rings as hydrophilic tails to guide the self-assembly of the SSS. The monochlorinated BODIPY core is the activatable unit based on thiol-halogen nucleophilic substitution of H_2_S. In the absence of H_2_S, the nanostructured photothermal agent (Nano-PT) produces minimal photothermal effects with absorption and emission at 540 and 589 nm, respectively. However, the H_2_S response results in high NIR absorption near 790 nm, which not only causes efficient photothermal energy conversion with 785 nm laser irradiation, but also produces bright luminescence in the NIR-II region (Fig. 10B). Using these excellent properties, the Nano-PT enables efficient photothermal ablation of imaging-guided colon cancer tumors (Fig. 10C).

An *et al.*^104^ designed an intelligent diagnostic reagent for colon cancer based on the *in situ* reaction of cuprous oxide (Cu_2_O) with endogenous H_2_S at the colon tumor site (Fig. 10D). Highly expressed endogenous H_2_S in colon tumors reacts with cuprous oxide and produces copper sulfide, which has strong near-infrared absorption, triggering photoacoustic and photothermal effects (Fig. 10E). The design of the *in situ* reaction at the tumor site reduces the damage to normal tissues during treatment and produces a significant therapeutic effect (Fig. 10F).

## 4. Summary and outlook

Abnormalities in H_2_S levels are associated with the development of a variety of diseases, such as colon cancer, breast cancer and ovarian cancer. In order to achieve early prevention and diagnosis of related diseases, research aimed at producing highly sensitive and selective H_2_S probes has been promoted. Among the possible techniques available, optical detection methods have higher sensitivity than traditional H_2_S detection methods. The transition from a single wavelength fluorescent probe to a more sensitive ratiometric fluorescent probe reduces the effects of external environment and other factors. In order to achieve real-time monitoring of H_2_S *in vivo*, further development from a short-wavelength fluorescent probe to a second near-infrared fluorescent probe, and photoacoustic probe with high tissue penetration has taken place. More importantly, utilizing the special microenvironment with high expression of endogenous H_2_S at the colon tumor site, H_2_S-activated intelligent therapeutic agents have been developed. Compared with using the traditional reagents, this strategy reduces the damage to normal tissues and shows more obvious therapeutic effects. Although many H_2_S probes with high sensitivity and high selectivity have been developed so far, as well as H_2_S smart reagents for cancer treatment, it is still necessary to continue to explore probes with lower side effects before their clinical application.

We believe that the integration of diagnostic and therapeutic agents for H_2_S detection and related disease treatment has a broad development prospect. In our subsequent research we aim to: (i) develop diagnostic reagents that are easy to prepare, and have good stability and biocompatibility; (ii) combine a variety of methods for tumor diagnosis and treatment, to develop intelligent diagnostic reagents with multi-modal diagnosis and synergistic treatment—for example, combining fluorescent probes with photoacoustic probes;^105^ (iii) undertake an in-depth study of the side effects of various agents, as well as their potential toxicity. Only once the problems described in this review have been solved, can the reagents can be further applied to clinical use.

## Conflicts of interest

There are no conflicts of interests to declare.

## Supplementary Material

## References

[cit1] Abe K., Kimura H. (1996). J. Neurosci..

[cit2] Hosoki R., Matsuki N., Kimura H. (1997). Biochem. Biophys. Res. Commun..

[cit3] Szabo C. (2007). Nat. Rev. Drug Discovery.

[cit4] Cao X., Wu Z., Xiong S., Cao L., Sethi G., Bian J. S. (2018). Biochem. Pharmacol..

[cit5] Chen Y. H., Yao W. Z., Ding Y. L., Geng B., Lu M., Tang C. S. (2008). Pulm. Pharmacol. Ther..

[cit6] Elsey D. J., Fowkes R. C., Baxter G. F. (2010). Cell Biochem. Funct..

[cit7] Li L., Rose P., Moore P. K. (2011). Annu. Rev. Pharmacol. Toxicol..

[cit8] Han Y., Qin J., Chang X., Yang Z., Du J. (2006). Cell. Mol. Neurobiol..

[cit9] Kimura H. (2011). Exp. Physiol..

[cit10] Kabil O., Banerjee R. (2010). J. Biol. Chem..

[cit11] Singh S., Padovani D., Leslie R. A., Chiku T., Banerjee R. (2009). J. Biol. Chem..

[cit12] Kaushik R., Ghosh A., Jose D. A. (2016). J. Lumin..

[cit13] Hartle M. D., Pluth M. D. (2016). Chem. Soc. Rev..

[cit14] Wang R. (2012). Physiol. Rev..

[cit15] Lefer D. J. (2007). Proc. Natl. Acad. Sci. U. S. A..

[cit16] Blackstone E., Morrison M., Roth M. B. (2005). Science.

[cit17] Zanardo R. C., Brancaleone V., Distrutti E., Fiorucci S., Cirino G., Wallace J. L. (2006). FASEB J..

[cit18] Wallace J. L. (2007). Trends Pharmacol. Sci..

[cit19] Eto K., Asada T., Arima K., Makifuchi T., Kimura H. (2002). Biochem. Biophys. Res. Commun..

[cit20] Zhao W., Zhang J., Lu Y., Wang R. (2001). EMBO J..

[cit21] Choi M. G., Cha S., Lee H., Jeon H. L., Chang S.-K. (2009). Chem. Commun..

[cit22] Jimenez D., Martinez-Manez R., Sancenon F., Ros-Lis J. V., Benito A., Soto J. (2003). J. Am. Chem. Soc..

[cit23] Furne J., Saeed A., Levitt M. D. (2008). Am. J. Physiol.: Regul., Integr. Comp. Physiol..

[cit24] Ishigami M., Hiraki K., Umemura K., Ogasawara Y., Ishii K., Kimura H. (2009). Antioxid. Redox Signaling.

[cit25] Zhang C., Wei L., Wei C., Zhang J., Wang R., Xi Z., Yi L. (2015). Chem. Commun..

[cit26] Shen X., Pattillo C. B., Pardue S., Bir S. C., Wang R., Kevil C. G. (2011). Free Radical Biol. Med..

[cit27] Que E. L., Domaille D. W., Chang C. J. (2008). Chem. Rev..

[cit28] Thoumine O., Ewers H., Heine M., Groc L., Frischknecht R., Giannone G., Poujol C., Legros P., Lounis B., Cognet L., Choquet D. (2008). Chem. Rev..

[cit29] Yang Y., Zhao Q., Feng W., Li F. (2013). Chem. Rev..

[cit30] Hua B., Shao L., Yu G., Huang F. (2016). Chem. Commun..

[cit31] Jung H. S., Verwilst P., Kim W. Y., Kim J. S. (2016). Chem. Soc. Rev..

[cit32] Dong B., Song X., Wang C., Kong X., Tang Y., Lin W. (2016). Anal. Chem..

[cit33] Dong B., Song X., Kong X., Wang C., Tang Y., Liu Y., Lin W. (2016). Adv. Mater..

[cit34] Lippert A. R., New E. J., Chang C. J. (2011). J. Am. Chem. Soc..

[cit35] Montoya L. A., Pluth M. D. (2012). Chem. Commun..

[cit36] Yu C., Li X., Zeng F., Zheng F., Wu S. (2013). Chem. Commun..

[cit37] Wang R., Yu F., Chen L., Chen H., Wang L., Zhang W. (2012). Chem. Commun..

[cit38] Wu M.-Y., Li K., Hou J.-T., Huang Z., Yu X.-Q. (2012). Org. Biomol. Chem..

[cit39] Wang J., Long L., Xie D., Zhan Y. (2013). J. Lumin..

[cit40] Wang M.-Q., Li K., Hou I.-T., Wu M.-Y., Huang Z., Yu X.-Q. (2012). J. Org. Chem..

[cit41] Hou F., Cheng J., Xi P., Chen F., Huang L., Xie G., Shi Y., Liu H., Bai D., Zeng Z. (2012). Dalton Trans..

[cit42] Liu J., Sun Y.-Q., Zhang J., Yang T., Cao J., Zhang L., Guo W. (2013). Chem.–Eur. J..

[cit43] Cao X., Lin W., Zheng K., He L. (2012). Chem. Commun..

[cit44] Liu T., Xu Z., Spring D. R., Cui J. (2013). Org. Lett..

[cit45] Filipovic M. R., Zivanovic J., Alvarez B., Banerjee R. (2018). Chem. Rev..

[cit46] Szabo C. (2007). Nat. Rev. Drug Discovery.

[cit47] Wu D., Si W., Wang M., Lv S., Ji A., Li Y. (2015). Nitric Oxide.

[cit48] Wu D., Li M., Tian W., Wang S., Cui L., Li H., Wang H., Ji A., Li Y. (2017). Sci. Rep..

[cit49] Cai W. J., Wang M. J., Ju L. H., Wang C., Zhu Y. C. (2010). Cell Biol. Int..

[cit50] Monsuez J. J., Charniot J. C., Vignat N., Artigou J. Y. (2010). Int. J. Cardiol..

[cit51] Bouwman P., Jonkers J. (2012). Nat. Rev. Cancer.

[cit52] Greco F., Vicent M. J. (2009). Adv. Drug Delivery Rev..

[cit53] Aceto N., Bardia A., Miyamoto D. T., Donaldson M. C., Wittner B. S., Spencer J. A., Yu M., Pely A., Engstrom A., Zhu H., Brannigan B. W., Kapur R., Stott S. L., Shioda T., Ramaswamy S., Ting D. T., Lin C. P., Toner M., Haber D. A., Maheswaran S. (2014). Cell.

[cit54] Yan Q., Sang W. (2016). Chem. Sci..

[cit55] Huang X., Liu H., Zhang J., Xiao B., Wu F., Zhang Y., Tan Y., Jiang Y. (2019). New J. Chem..

[cit56] Zhang H., Chen J., Xiong H., Zhang Y., Chen W., Sheng J., Song X. (2019). Org. Biomol. Chem..

[cit57] Qian Y., Zhang L., Ding S., Deng X., He C., Zheng X. E., Zhu H.-L., Zhao J. (2012). Chem. Sci..

[cit58] Tian X., Li Z., Lau C., Lu J. (2015). Anal. Chem..

[cit59] Liu Y., Meng F., He L., Liu K., Lin W. (2016). Chem. Commun..

[cit60] Zhou X., Lee S., Xu Z., Yoon J. (2015). Chem. Rev..

[cit61] Chang M. J., Kim K., Kang C., Lee M. H. (2019). ACS Omega.

[cit62] Wang F., Xu G., Gu X., Wang Z., Wang Z., Shi B., Lu C., Gong X., Zhao C. (2018). Biomaterials.

[cit63] Thorson M. K., Majtan T., Kraus J. P., Barrios A. M. (2013). Angew. Chem., Int. Ed..

[cit64] Chen B., Li W., Lv C., Zhao M., Jin H., Jin H., Du J., Zhang L., Tang X. (2013). Analyst.

[cit65] Bailey T. S., Pluth M. D. (2013). J. Am. Chem. Soc..

[cit66] Qu X., Li C., Chen H., Mack J., Guo Z., Shen Z. (2013). Chem. Commun..

[cit67] Liu C., Pan J., Li S., Zhao Y., Wu L. Y., Berkman C. E., Whorton A. R., Xian M. (2011). Angew. Chem., Int. Ed..

[cit68] Peng H., Cheng Y., Dai C., King A. L., Predmore B. L., Lefer D. J., Wang B. (2011). Angew. Chem., Int. Ed..

[cit69] Sasakura K., Hanaoka K., Shibuya N., Mikami Y., Kimura Y., Komatsu T., Ueno T., Terai T., Kimura H., Nagano T. (2011). J. Am. Chem. Soc..

[cit70] Chen Y., Zhu C., Yang Z., Chen J., He Y., Jiao Y., He W., Qiu L., Cen J., Guo Z. (2013). Angew. Chem., Int. Ed..

[cit71] Bae S. K., Heo C. H., Choi D. J., Sen D., Joe E. H., Cho B. R., Kim H. M. (2013). J. Am. Chem. Soc..

[cit72] Song G., Liu A., Jiang H., Ji R., Dong J., Ge Y. (2019). Anal. Chim. Acta.

[cit73] Youssef S., Zhang S., Ai H.-w. (2019). ACS Sens..

[cit74] Hong Y., Zhang P., Wang H., Yu M., Gao Y., Chen J. (2018). Sens. Actuators, B.

[cit75] Wang C., Ding Y., Bi X., Luo J., Wang G., Lin Y. (2018). Sens. Actuators, B.

[cit76] Zhao C., Zhang X., Li K., Zhu S., Guo Z., Zhang L., Wang F., Fei Q., Luo S., Shi P., Tian H., Zhu W. H. (2015). J. Am. Chem. Soc..

[cit77] Feng X., Zhang T., Liu J. T., Miao J. Y., Zhao B. X. (2016). Chem. Commun..

[cit78] Sancataldo G., Silvestri L., Mascaro A. L. A., Sacconi L., Pavone F. S. (2019). Optica.

[cit79] Jemielita M., Taormina M. J., DeLaurier A., Kimmel C. B., Parthasarathy R. (2013). J. Biophotonics.

[cit80] Hammers M. D., Taormina M. J., Cerda M. M., Montoya L. A., Seidenkranz D. T., Parthasarathy R., Pluth M. D. (2015). J. Am. Chem. Soc..

[cit81] Du Z., Zhang R., Song B., Zhang W., Wang Y.-L., Liu J., Liu C., Xu Z. P., Yuan J. (2019). Chem.–Eur. J..

[cit82] Urriza-Arsuaga I., Bedoya M., Orellana G. (2019). Anal. Chem..

[cit83] Du Z., Song B., Zhang W., Duan C., Wang Y. L., Liu C., Zhang R., Yuan J. (2018). Angew. Chem., Int. Ed..

[cit84] Tang Y., Li Y., Hu X., Zhao H., Ji Y., Chen L., Hu W., Zhang W., Li X., Lu X., Huang W., Fan Q. (2018). Adv. Mater..

[cit85] Xu G., Yan Q., Lv X., Zhu Y., Xin K., Shi B., Wang R., Chen J., Gao W., Shi P., Fan C., Zhao C., Tian H. (2018). Angew. Chem., Int. Ed..

[cit86] Pu K., Shuhendler A. J., Jokerst J. V., Mei J., Gambhir S. S., Bao Z., Rao J. (2014). Nat. Nanotechnol..

[cit87] Chen K., Zhang B., Liu S., Yu Q. (2019). Sens. Actuators, B.

[cit88] Shi B., Gu X., Fei Q., Zhao C. (2017). Chem. Sci..

[cit89] Ma T., Zheng J., Zhang T., Xing D. (2018). Nanoscale.

[cit90] Guo B., Sheng Z., Hu D., Liu C., Zheng H., Liu B. (2018). Adv. Mater..

[cit91] Guo B., Chen J., Chen N., Middha E., Xu S., Pan Y., Wu M., Li K., Liu C., Liu B. (2019). Adv. Mater..

[cit92] Viale M., Vecchio G., Monticone M., Bertone V., Giglio V., Maric I., Cilli M., Bocchini V., Profumo A., Ponzoni M., Emionite L., Rocco M. (2019). Pharm. Res..

[cit93] Zheng M., Liu S., Guan X., Xie Z. (2015). ACS Appl. Mater. Interfaces.

[cit94] Thirumalaivasan N., Venkatesan P., Lai P.-S., Wu S.-P. (2019). ACS Appl. Bio Mater..

[cit95] Zhang H., Kong X., Tang Y., Lin W. (2016). ACS Appl. Mater. Interfaces.

[cit96] Chen W., Zhang Y., Li X., Chen H., Sun J., Feng F. (2017). ACS Appl. Mater. Interfaces.

[cit97] Momma T., Hamblin M. R., Wu H. C., Hasan T. (1998). Cancer Res..

[cit98] Nathel H. (1998). Appl. Opt..

[cit99] Ma Y., Li X., Li A., Yang P., Zhang C., Tang B. (2017). Angew. Chem., Int. Ed..

[cit100] Wu L., Sun Y., Sugimoto K., Luo Z., Ishigaki Y., Pu K., Suzuki T., Chen H. Y., Ye D. (2018). J. Am. Chem. Soc..

[cit101] Wang R., Dong K., Xu G., Shi B., Zhu T., Shi P., Guo Z., Zhu W.-H., Zhao C. (2019). Chem. Sci..

[cit102] Robinson J. T., Tabakman S. M., Liang Y., Wang H., Casalongue H. S., Vinh D., Dai H. (2011). J. Am. Chem. Soc..

[cit103] Shi B., Yan Q., Tang J., Xin K., Zhang J., Zhu Y., Xu G., Wang R., Chen J., Gao W., Zhu T., Shi J., Fan C., Zhao C., Tian H. (2018). Nano Lett..

[cit104] An L., Wang X., Rui X., Lin J., Yang H., Tian Q., Tao C., Yang S. (2018). Angew. Chem., Int. Ed..

[cit105] Guo B., Feng Z., Hu D., Xu S., Middha E., Pan Y., Liu C., Zheng H., Qian J., Sheng Z., Liu B. (2019). Adv. Mater..

